# Fluorescence lateral flow competitive protein binding assay for the assessment of serum folate concentrations

**DOI:** 10.1371/journal.pone.0217403

**Published:** 2019-06-05

**Authors:** Elizabeth G. Rey, Julia L. Finkelstein, David Erickson

**Affiliations:** 1 Sibley School of Mechanical and Aerospace Engineering, Cornell University, Ithaca, New York, United States of America; 2 Division of Nutritional Sciences, Cornell University, Ithaca, New York, United States of America; University of Houston, UNITED STATES

## Abstract

Folate is a micronutrient required for the production of new cells, making it a key factor in early fetal development and ensuring normal growth and maintenance of health. The increase in consumption of folate due to increased periconceptional supplementation and fortification of grains in many countries has led to a decrease in occurrence of folate deficiency and a class of birth defects called neural tube defects. However, an opportunity remains to further improve folate status of populations in areas with limited access to fortified foods and supplementation. Screening of women of reproductive age and other vulnerable populations for folate status would increase our understanding of the magnitude of the burden of folate deficiency and inform monitoring of public health programs. Current gold standard methods for folate assessment are time-intensive and require cold chain, sophisticated laboratory infrastructure, and highly-trained personnel. Our lateral flow assay is low-cost, easy to use, and allows a user to assess folate insufficiency at the point of care in less than 40 minutes. We evaluated the sensitivity and specificity of our assay in 24 human serum samples, including 8 samples with folate concentrations less than 10.0 nmol/L and 14 samples less than 13.4 nmol/L using the Immulite 2000 commercial assay as a reference standard. The sensitivity and specificity were found to be 93% (95% CI: 54.7–100.0) and 91% (95% CI: 80.0–100.0), respectively, when using our test to determine folate insufficiency based on a cutoff of 13.4 nmol/L. Our point-of-care diagnostic test for folate concentrations could inform screening and public health programs in at-risk populations.

## Introduction

Folate is a B-vitamin which is essential for DNA synthesis and cellular division[1], including fetal growth and development[2]. Factors that can lead to insufficient folate status include inadequate intake, increased requirements (e.g., pregnancy), conditions which inhibit absorption, and antifolate medications (e.g., pyrimethamine, methotrexate)[3]. Folate deficiency can lead to megaloblastic anemia, which can cause symptoms of weakness, fatigue, and shortness of breath[3]. Folate requirements are increased during pregnancy and lactation, to support cell replication in fetal, placental, and maternal tissues; inadequate maternal folate status during pregnancy has been associated with increased risk of adverse pregnancy outcomes, including pregnancy loss, low birth weight, and premature delivery[3].

Many factors have been shown to play a role in pregnancy outcomes[4,5], however, maternal periconceptional supplementation with folic acid (FA, i.e., the synthetic form of the vitamin) has been shown to decrease the risk of a class of birth defects called neural tube defects (NTDs)[6–9]. These birth defects, including spina bifida and anencephaly, are a leading cause of neonatal morbidity and mortality worldwide, with medical costs of almost $300,000 per person born with spina bifida, and can cause permanent paralysis and lifelong disability[2]. Periconceptional folic acid supplementation has been shown to reduce the risk and recurrence of NTDs by 50 to 70%,[10,11] however, the closure of the neural tube within 28 days of conception means that by the time that most women know that they are pregnant, it is too late to begin FA supplementation for NTD prevention[11]. For this reason, it is critical that women who are at risk for becoming pregnant already have sufficient folate status. This has been a driving force stimulating the mandatory FA fortification of grains in over 80 countries[10,12]. In the United States, FA fortification decreased the prevalence of spina bifida and anencephaly by 31 and 16 percent, respectively, in the first year after fortification was mandated[6]. However, according to the Centers for Disease Control and Prevention (CDC), FA supplementation and food fortification programs could still prevent 150,000 to 210,000 NTDs each year out of approximately 300,000 total worldwide. The World Health Organization (WHO) has called for further research towards less invasive methods for folate assessment as well as surveillance systems for the assessment of folate status in women of reproductive age[13]. Prevalence of elevated blood concentrations of folate have also increased as a result of folate fortification[14], leading to a need to monitor populations for excess supplementation with FA.

Folate status is typically measured as total folate concentration, including the primary form in circulation, 5-methyltetrahydrofolate (5-MTHF), as well as other oxidized and reduced forms[3]. It is measured in serum or plasma and in erythrocytes or red blood cells (RBCs). Erythrocyte folate concentrations are a biomarker of longer term folate status, over the lifetime of circulating RBCs, approximately 90 to 120 days, whereas serum folate reflects recent folate intake[15]. Serum or plasma folate is the more commonly measured biomarker, particularly in population-based surveillance programs, due to its comparatively lower complexity and cost of laboratory analysis[15].

Folate deficiency and sufficiency have been defined based on cut-offs for two different physiological indications: megaloblastic anemia and elevated serum or plasma homocysteine concentrations[16]. The WHO uses the following cutoffs for serum folate concentrations based on risk of megaloblastic anemia: folate deficiency at less than 6.8 nmol/L, possible deficiency between 6.8 and 13.4 nmol/L, and normal folate between 13.5 and 45.3 nmol/L[16]. Cutoffs based on elevated homocysteine concentrations denote folate deficiency as less than 10 nmol/L, with all concentrations above this indicating sufficient folate status[16]. Folate concentrations above 45.3 nmol/L indicate elevated folate status, although the implications of concentrations greater than 45.3 nmol/L are not well understood or agreed upon[17].

Currently, there are three main laboratory-based methods to determine folate status: a microbiological assay (MA), a competitive protein binding assay, and liquid chromatography-tandem mass spectrometry (LC-MS/MS). The microbiological assay uses the bacteria *L*. *casei* and its growth in the presence of folate to determine folate concentrations, using an antioxidant such as L-ascorbic acid to prevent folate degradation over time[18]. The competitive protein binding assay (CPBA) uses the affinity between folate and a transport protein called folate binding protein (FBP) to determine folate concentrations[19]. These two assays measure total folate, which includes all the oxidized and reduced forms. LC-MS/MS can differentiate between 5-MTHF and other forms of folate. All of these methods require cold chain, sophisticated laboratory infrastructure, and highly-trained personnel, and are time-intensive—ranging from 3 to 48 hours to complete. For these methods, a venous blood sample is collected and either analyzed immediately or processed and frozen until analyses can be performed. Sample volumes for typical CPBAs range from 50 to 400 μL of serum per test[20–22]. Sample preparation steps to separate endogenous FBP from folate in the sample are required in CPBA and LC-MS/MS protocols[20–22]. This need for laboratory infrastructure to measure folate constrains assessment of folate status in many clinical and field settings. This is one reason that the magnitude of folate deficiency around the world is largely unknown[12].

The development of a point-of-care test for assessment of serum folate concentrations presents several challenges due to the nature of folate in serum. First, endogenous FBP in human serum is bound with high affinity to a portion of the folate in circulation[23,24]. This FBP is typically denatured or separated from bound folate in commercial CPBAs by chemical denaturation. The chemicals used in this step (i.e., dithiothreitol, potassium hydroxide (KOH), potassium cyanide)[20–22] are either unstable at room temperature or toxic, making them unsuitable for use at the point of care. Alternatively, FBP can be denatured through a heating step. Although heat denaturation can be at least partially reversible, heating to 100°C has been used to denature FBP irreversibly in some methods (e.g., Bio-Rad Quantaphase II Folate/Vitamin B_12_ Radioassay Kit)[20,25].

Our lateral flow assay (LFA) is a CPBA with fluorescent labels that can be imaged and analyzed by a portable fluorescent imaging device to quantify folate concentration in serum. This test requires only 20 μL of serum and can be completed in less than 40 minutes. Although some sample pre-processing is necessary to ensure reliable test results, any equipment can be used to heat the sample to 100°C, and no further laboratory equipment is required.

## Materials and methods

### Lateral flow strip writing and assembly

Nitrocellulose (NC) membranes with plastic backing were purchased from EMD Millipore (HF180MC100, Billerica, MA, USA). Lines were written onto the NC membrane using an Automated Lateral Flow Reagent Dispenser (Claremont Bio Solutions, Upland, CA, USA). Goat polyclonal anti-fluorescein isothiocyanate (FITC) antibodies (ab19224, Abcam, Inc, Cambridge, MA, USA) raised against FITC conjugated to KLH were dispensed onto the membrane as a control line at a concentration of 0.5 mg/ml in 1.9mM borate buffer with 3% (w/v) trehalose. FBP from bovine milk (Scripps Laboratories, San Diego, CA, USA) was dispensed onto the membrane as a test line at a concentration of 0.9 mg/ml in 1.55 mM borate buffer. After dispensing, NC membranes were placed in a desiccated incubator at 37°C to dry overnight. After drying, membranes were kept at room temperature in a desiccator (<20% relative humidity).

**Fig 1:**
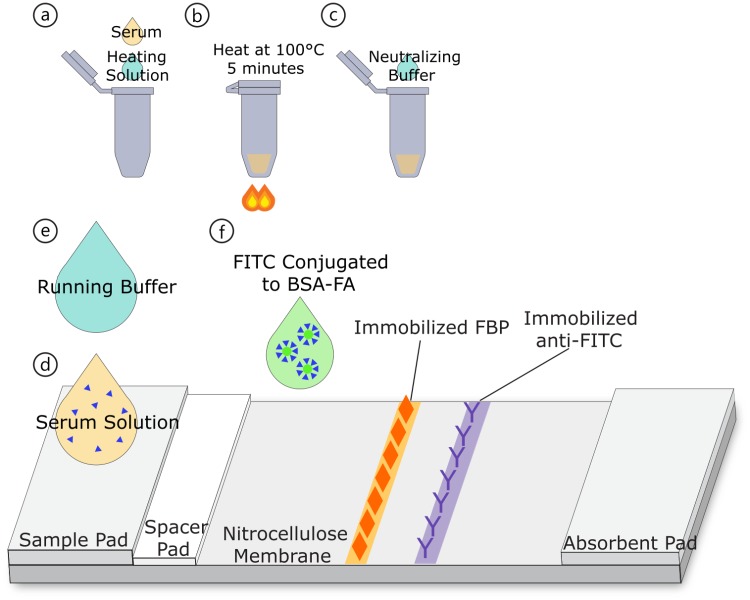
Schematic of sample processing and lateral flow assay. (a) Combination of serum sample with high-pH buffer. (b) Heating of serum solution. (c) Addition of acidic buffer to lower pH. (d) Application of prepared serum solution to LFA. (e) Application of running buffer. (f) Addition of FITC conjugates to nitrocellulose membrane.

Lateral flow cards were assembled using glass fiber and cellulose fiber pads on the adhesive-backed NC membranes, as shown in Fig 1. Glass fiber conjugate pads and cellulose fiber sample pads (GFDX103000 and CFSP203000, respectively) were also purchased from EMD Millipore. Glass fiber conjugate pads were cut in half to be 5 mm by 30 mm and placed such that there was ~1 mm overlap with the NC membrane. Cellulose fiber sample pads were used as an absorbent pad upstream of the NC membrane and as a sample pad. The sample pad overlapped with the glass fiber pad by ~2 mm and was trimmed to be 12 mm by 30 mm. Once the lateral flow cards were assembled, they were cut to individual 4 mm strips.

FITC-FA conjugates were made using Lightning Link Conjugation Kits (Expedeon Ltd, Cambridge, UK) and FA-BSA conjugates (Fitzgerald Industries, North Acton, MA, USA). These conjugates were stored 1:200 in a conjugate buffer at 4°C. The conjugate buffer consisted of 2mM borate buffer with 5% (w/v) sucrose, 1% (w/v) bovine serum albumin (BSA), 0.5% (v/v) Tween20, and 0.02% (w/v) sodium azide.

### Serum sample preparation

Calibration curves made with human serum spiked with FA and 5-MTHF were performed as part of a previous work[26]. Human serum samples of varying folate concentration were purchased from Discovery Life Sciences Inc. (Los Osos, CA, USA). Serum folate concentrations were determined using the Immulite 2000 Folic Acid CPBA according to the given assay protocol. The Immulite was chosen as the reference standard because of its similarities in assay formulation to our LFA. Both are CPBAs and should be able to deliver similar results. Of the 24 samples used to validate this assay, 8 samples had folate concentrations less than 10 nmol/L and 12 less than 13.4 nmol/L. Samples were chosen from a list of samples with measured folate concentrations and the authors chose samples with varying concentrations in order to validate the assay over the widest range possible. No samples with known concentrations less than 6.8 nmol/L were available for purchase. The sample demographics included 17 female and 7 male participants, with a mean age of 56.4 years and an age range of 24 to 89 years.

In preparation for running on the LFA, human serum samples were mixed with a high-pH solution. The sample preparation procedure is shown in Fig 1(a)–1(c). The high-pH solution was prepared immediately prior to use and consists of 60 mM KOH and 21.3 mM L-ascorbic acid in deionized water. For each test, 20 μL of human serum was mixed with 30 μL of the high-pH solution. The two liquids were dropped into a microcentrifuge tube and the tube was shaken side to side 2 to 3 times to ensure mixing. This solution has a pH near 11 and prevents the proteins in the serum from coagulation during heating[27]. The ascorbic acid in the solution serves to prevent oxidation of folate during heating and exposure to light[28,29].

After mixing with the high-pH solution, the serum was heated to 100°C for 5 minutes in an electric dry bath (VWR, Radnor, PA, USA). The tubes were removed from the heat and allowed to cool at room temperature for 5 minutes. Heating to boiling temperature denatures the endogenous FBP in the sample, which releases the bound folate[20,25]. The pH of the solution was brought to ~9 by the addition and mixing in of 7 μL of a dilute acetate buffer (0.5% (v/v), Acetate Buffer Solution pH 4.0, BDH/VWR Analytical). At a pH of around 9.3, the affinity of FBP to FA and 5-MTHF is very similar[30]. Bringing the sample to near this pH allows us to detect both with the same affinity, making it easier to compare serum samples to FA standards. The sample is now ready for application to the LFA.

For the samples tested without heat, the same amounts of high-pH solution and serum were combined and allowed to rest at room temperature for 10 minutes. To achieve a pH around 9, we added 10 μL of the dilute acetate buffer and mixed the solution.

### Lateral flow assay procedure

The LFA procedure is indicated in Fig 1(d)–1(f). To begin the running of the LFA, 40 μL of the prepared serum solution was dropped onto the sample pad of the lateral flow strip. Immediately after this, 30 μL of a running buffer was added to the sample pad. The running buffer consisted of 1x tris-buffered saline with 1% (w/v) BSA, 1.5% Tween20, and 0.02% sodium azide. These solutions were allowed to flow across the NC membrane for 7 minutes, before another 40 μL of running buffer was added to the sample pad as a wash step. After another 8 minutes, 10 μL of FITC-FA conjugates diluted 1:1.5 in running buffer was added to the NC membrane just upstream of the glass fiber conjugate pad. The glass fiber pad serves to prevent flow of the conjugates into the cellulose fiber sample pad, where they could become fixed. The strips were imaged in a portable fluorescent imaging device 9 minutes after the FITC conjugates were added. In total, the strips were imaged 24 minutes after the addition of the serum sample. Including sample preparation, the total time for the assay is less than 40 minutes.

As the serum and running buffer solution flowed across the NC membrane, the folate in the sample could interact with the FBP immobilized on the surface. Interfering components of the serum solution were then washed off the membrane during the running buffer wash step. This ensured that with the addition of the FITC-FA conjugates there was minimal interaction between the conjugates and components in the serum. The FITC-FA conjugates interacted with the free FBP on the test line, with the FA binding to FBP with the same high affinity as endogenous folate at a pH near 9. With increased concentration of folate in the serum sample, the fluorescent intensity of the test line decreased. The remaining FITC-FA conjugates flowed past the test line and bound to the anti-FITC immobilized on the control line.

For tests run with a traditional LFA format, we used a lateral flow strip with an extra 5 mm wide glass fiber conjugate pad placed adjacent to the spacer pad. Onto this conjugate pad we pipetted 4 μL of the FITC-FA conjugates. We then added 40 μL of the serum solution to the sample pad, followed by 30 μL of running buffer. These were allowed to flow for 15 minutes before imaging.

### Imaging device and Image processing

To determine the signal from this fluorescent lateral flow strip, we used a portable imaging device designed previously by our lab. This device was described previously by Lu et al[31]. Briefly, it consists of a Raspberry Pi computer board, a 5-megapixel CMOS camera, a lithium-ion battery, blue LEDs, fluorescence and focusing optics, and a light-tight 3D-printed case with a tray for the lateral flow strip cassette. This device can be prompted to take an image with a web browser on any Wi-Fi-enabled smart device, and once the image is collected it is sent via Wi-Fi to the smart device. This image file contained raw Bayer data, and was de-mosaiced, cropped, and analyzed by Python scripts. The output signal that we used from each image was the ratio of the intensity of the test and control lines, called the T/C ratio.

### Curve fit and receiver operating characteristics curve analysis

A four-parameter logistic curve fit for the triplicate results on 24 samples was performed using Python. The correlation coefficient was calculated using a residual analysis. ROC curves were determined empirically using the *pROC* package in RStudio. Determination of folate status was made based on folate concentration as indicated by Immulite. AUCs were determined using trapezoids[32], and confidence intervals for the AUCs were computed using the DeLong method[33]. Confidence intervals for sensitivity and specificity values were determined using the bootstrap method.

## Results and discussion

### Human serum results

**Fig 2:**
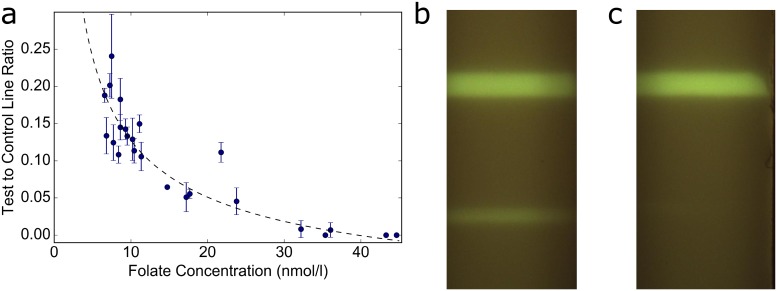
Human serum results. (a) Mean T/C ratio versus folate concentration for 24 human serum samples. Error bars shown are standard deviation, n = 3. Dotted line shows four-parameter logistic curve fit. (b,c) Images of fluorescent signal from test strip for low and high folate concentration serum, respectively.

A total of 24 archived human serum samples with folate concentrations ranging from 6.8 nmol/L to 44.8 nmol/L were analyzed in triplicate. The T/C ratio, a commonly used metric in the quantification of LFAs[34–38], was determined for each LFA strip. The T/C ratios from each test are presented in Fig 2(a). The data was fitted with a 4-parameter logistic curve and demonstrated high correlation for curve fit (*r* = 0.87). Images of test strips taken using our portable fluorescence imaging device are shown in Fig 2(b) and 2(c). Fig 2(b) and 2(c) are examples of tests conducted with samples with lower and higher folate concentrations, respectively.

**Fig 3:**
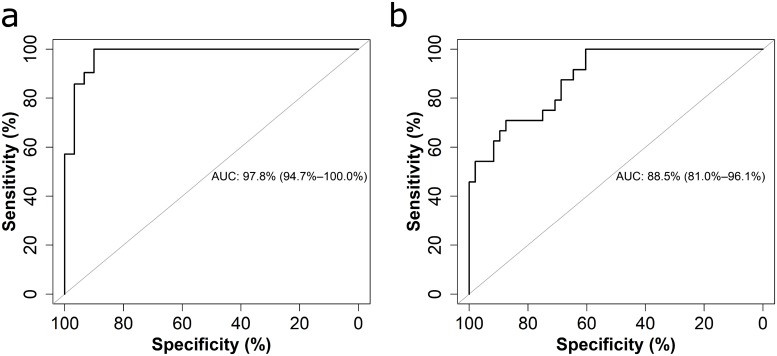
Receiver operating characteristics curves for two different cutoff concentrations. (a) Cutoff at 13.4 nmol/L folate concentration. (b) Cutoff at 10 nmol/L folate concentration.

To examine the diagnostic ability of this LFA to predict folate deficiency, we analyzed this data using receiver operating characteristic (ROC) curves. Since none of the samples had folate concentrations less than 6.8 nmol/L, we considered cutoffs of 13.4 nmol/L (possible deficiency) and 10.0 nmol/L (folate deficiency) to generate the ROC curves. The ROC curve for determination of possible deficiency (cutoff at 13.4 nmol/L) is shown in Fig 3(a), with a sensitivity and specificity of 93% (95% CI: 54.7–100.0) and 91% (95% CI: 80.0–100.0), respectively. The curve for determination of folate deficiency (cutoff at 10.0 nmol/L) is shown in Fig 3(b), with sensitivity and specificity values at 87.5% (95% CI: 58.3–100.0) and 68.8% (95% CI: 52.08–87.5), respectively.

We also examined the area under the curve (AUC), as a summary measure of the accuracy of a diagnostic test[33]. This indicates the probability that given two randomly selected participants, one with higher folate status and one with lower folate status, the results will indicate that the participant with higher folate status has a lower T/C ratio than the one with lower folate status. The AUC was 97.8% for the 13.4 nmol/L cutoff and 88.5% for the 10.0 nmol/L cut-off. The lower AUC for the 10.0 nmol/L cutoff is due in part to the close proximity of samples to the cutoff concentration, and the smaller number of samples below 10.0 nmol/L compared to 13.4 nmol/L, giving fewer opportunities to predict folate deficiency. Overall, the LFA results demonstrated high accuracy for the determination of possible folate deficiency. Although we were not able to test samples with concentrations less than 6.8 nmol/L, the high slope of the curve fit at low folate concentrations suggests that we would be able to quantify even lower sample concentrations. This LFA would not currently be able to quantify concentrations greater than 45 nmol/L, however it could be used as a qualitative test for folate greater than 45 nmol/L or adapted to measure higher concentrations for populations where screening for elevated serum folate is necessary.

### Results without sample heating

**Fig 4:**
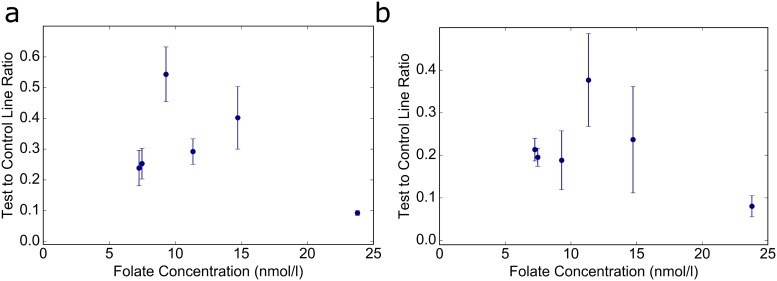
Serum results without heat denaturation and without washing and secondary addition of FITC conjugates. (a) Mean T/C ratio versus folate concentration for 6 serum samples tested without heating. Error bars shown are standard deviation, n = 3. (b) Mean T/C ratio versus folate concentration for 6 serum samples tested in a traditional lateral flow assay format. Error bars shown are standard deviation, n = 3.

Sample preparation is required to separate endogenous FBP from folate in circulation and prevent binding of endogenous FBP to test components. We used heat to irreversibly denature the FBP in the sample without impacting the proteins and antibodies on the LFA. The procedures for sample preparation are depicted in Fig 1(a)–1(c). When the LFA test was conducted without a heating step, it produced widely ranging and non-correlated T/C ratios. Fig 4(a) displays the results of 6 samples tested with a procedure nearly identical to our final protocol, excluding the 5-minute heating step.

Denaturation through heating is advantageous at the point of care, as it avoids the use of toxic or unstable chemicals which could pose a threat to the user as well as interfere with the on-strip assay. However, heating of a serum sample also poses some challenges because of the heat-labile nature of some forms of folate, the tendency of serum to coagulate when heated above ~60°C, and the energy required to heat the sample to 100°C in a field setting. The propensity of some forms of folate, including 5-MTHF, to degrade when exposed to heat or light can be decreased by the addition of L-ascorbic acid[28,29]. The coagulation of serum proteins can be prevented through dilution and an increase in the pH of the solution, such as through the addition of a solution of KOH[27]. In a setting without access to electricity and a hot plate or dry bath, the sample could also be heated to 100°C for 5 minutes using boiling water. The necessity of this heating step may present a difficulty in the operation of this diagnostic in the field, and so the development of improved methods to implement this heating step in settings without access to electricity is a source for future work.

### Results with traditional LFA Architecture

Traditional LFAs typically contain antibody conjugates dried in a conjugate pad, often comprised of glass fiber[39]. This allows for dry storage of the conjugates on the strip and mixing of the conjugates with the sample as it flows through the conjugate pad. Although this method is useful in many applications of LFAs, in this study, allowing interaction between the FA conjugates and the serum sample interfered with the generation of signal at the test line. This interference occurred inconsistently in some serum samples and was not mitigated through heating of the sample. The setup of this LFA allows the serum to flow across the test line first, then washes away the remaining serum components on the strip before applying the FITC-BSA-FA solution. A schematic of our LFA is presented in Fig 1. Since the conjugates used are also non-traditional (i.e., a protein and small molecule rather than an antibody attached to the label), they do not require interaction with the folate in the sample. The labeled and unlabeled folate both can bind to the FBP on the test line at different times. The results we obtained using a traditional LFA setup for 6 serum samples are shown in Fig 4(b).

Although some of the samples resulted in T/C ratios that fit with the results expected *a priori*, results from several samples contained a wide range of T/C ratios at a single concentration and the average T/C ratio was much higher than expected at the given concentration. There were no correlations evident between serum folate concentration and T/C ratio from these tests. This LFA method introduces some additional steps for the user, including the placement of one drop of the FITC-BSA-FA solution onto the nitrocellulose membrane near the spacer pad. While additional steps required from a user does pose a possible limitation to this test, with an opaque metered dropper bottle, the solution could be stored in darkness to prevent photobleaching of the FITC, and application of the liquid to the strip would be relatively simple. This solution was stored at 4°C throughout these experiments, however, testing could be done in the future to determine its shelf life at room temperature. Future work could also include methods of drying the FITC solution or alternative application methods, as well as field testing of these improved methods. This field testing would be done along with standard methods for folate measurement to validate the accuracy of the LFA across the entire range of folate concentrations.

## Conclusions

This LFA platform could be used at the point of care and in field settings to detect concentrations of folate in serum. This assay delivers an accurate result regarding serum folate concentration in less than 40 minutes without the use of sophisticated laboratory equipment. Although sample preparation steps are required beyond that of traditional LFAs, we have developed a protocol which could be used in limited-resource settings. This point-of-care diagnostic method would inform screening for folate insufficiency in vulnerable populations, including women of reproductive age, and programs for anemia and birth defects prevention. This test could also inform assessment of folate status and representative population data worldwide. Along with appropriate folic acid supplementation and fortification programs, a folate diagnostic test at the point of care could help to reduce the burden of anemia and NTDs globally.

## Supporting information

S1 Dataset Raw data.zip. Raw data from lateral flow assays.
(ZIP)
